# Experimental Study on the Flexural Properties of Concrete Beams Reinforced with Hybrid Steel/Fiber-Belt-Bars

**DOI:** 10.3390/ma15103505

**Published:** 2022-05-13

**Authors:** Wenhu Gu, Hengrui Liu, Yun Dong

**Affiliations:** 1 Faculty of Architecture and Civil Engineering, Huaiyin Institute of Technology, Huai’an 223001, China; guwenhu@hyit.edu.cn (W.G.); dyun@hyit.edu.cn (Y.D.); 2 College of Water Conservancy and Hydropower Engineering, Hohai University, Xikang Road No. 1, Nanjing 210098, China

**Keywords:** fiber-belt-bar, reinforced concrete beam, mechanical property, flexural test, bearing capacity

## Abstract

Reinforcement corrosion poses a great threat to the safety of reinforced concrete structures, and the fiber-reinforced polymer is the ideal material to partially replace steel bars due to the high strength, light weight and good durability. However, the selection of appropriate fiber materials and a reasonable ratio of fiber bar to steel bar is not clear. Here, we measured the mechanical properties of fiber bars containing aramid fiber and carbon fiber. The deflection deformation, crack distribution and maximum crack width of the concrete upon various loads were experimentally and theoretically investigated. The predictions of the maximum crack width and deflection of reinforced concrete beams under various loads were proposed in ACI standard, which may provide guidance for further applications of fiber-belt-bar-containing concrete beams.

## 1. Introduction

Reinforcement corrosion is an important factor affecting the durability of reinforced concrete structures, and this poses a great threat to the safety of reinforced concrete (RC) structures [1,2,3]. Particularly, the corrosion in prestressed concrete bridge-beams provokes significant prestress losses, which induces cracking and excessive deflections during service conditions [4,5]. Fiber-reinforced polymer (FRP) is considered to be an ideal material to replace steel bars due to its high strength, light weight and good durability [6,7]. 

In the past few decades, FRP has been widely used in civil engineering, especially in some corrosive environments. However, the FRP bars are linear elastic materials with a low elastic modulus and brittle failure, which limits the further applications of FRP bars. For typical FRP materials, including carbon-fiber materials (CFRP), aramid-fiber materials (AFRP) and glass-fiber materials (GFRP), all have the weaknesses of linear elasticity, brittle failure and low elongation [8]. The ultimate strains of CFRP, AFRP and GFRP were 1.5%, 3% and 4%, respectively [9,10]. As a result, the ductility of current FRP bars and concrete structures was not ideal [11]. 

In recent years, mixtures of FRP and steel bars have been attempted to improve the stiffness and bearing capacity [12,13,14,15]. Tan et al. first proposed the concept of a replacement reinforcement ratio (*A_f_*/*A_s_*, *A_f_*: Cross-sectional area of fiber and *A_s_*: Cross-sectional area of steel) and found that mixed RC beams can provide sufficient bearing capacity when the tensile force provided by FRP bars does not exceed half of the total tensile force [16]. The effect of replacement reinforcement ratio (*A_f_*/*A_s_*) on the structural bearing capacity was also studied. 

Aiello et al., deduced that using sufficient reinforcement in FRP-RC beams can improve the bearing capacity and bending deformation of beams [17]. Leung and Balendran found that reducing the content of FRP can improve the ductility of concrete; however, the bearing capacity is insufficient [18]. More studies have been focused on the reasonable range of *A_f_*/*A_s_* ratio in mixed concrete beams. 

Qin et al. deduced that *A_f_*/*A_s_* in the range of 1 to 2.5 had high ductility and bearing capacity [19]. Pang et al. [20] suggested that the most reasonable value of *A_f_*/*A_s_* was in the range of 0.1–2.1. In further study of the mixed concrete beams, Qu et al. [21] calculated the average reinforcement ratio of beams by the equal-stiffness principle *E_s_A_s_* = *E_f_A_f_* and compared it with RC beams in ACI318-19 [22]. The relationship between the reinforcement ratio and failure modes of beams was proposed. The acceptability and debate of FRP were both increasing in the civil engineering field.

Here, we characterized performance of fiber-belt-bars containing aramid fiber and carbon fiber and their mixtures. The tensile reinforcements in the tensile zone were replaced by fiber-belt-bars in reinforced concrete beams, and the crack distribution and deflection deformation of the concrete beam under various vertical loads were studied. The theoretical calculations support our experimental results, which will contribute to the further development and wider applications of fiber-reinforced concretes.

## 2. Materials and Methods

### 2.1. Materials

**Figure 1:**
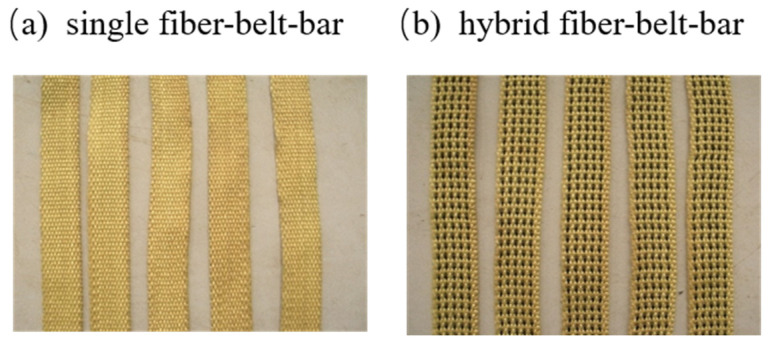
The photos of fiber-belt-bars.

The flexural behavior of RCs with single and hybrid fiber-belt-bars has been studied independently. In our experiments, aramid fiber (Kevlar29 and Kevlar49) and carbon fiber (T300) were selected because they possess excellent tensile properties in contrast to other fiber materials. In the four types of single fiber-belt-bars, AF-K29-1 and AF-K29-2 are made of Kevlar29 with actual cross-sectional areas of 16.5 and 25 mm^2^, respectively. AF-K49-1 and AF-K49-2 are made of Kevlar49 with actual cross-sectional areas of 16.5 and 25 mm^2^, respectively. In the two types of hybrid fiber-belt-bars, HAA1 is made of 80% Kevlar29, 10% Kevlar49 and 10% T300. HAC1 contains 80% Kevlar29 and 20% T300. The actual cross-sectional area of both hybrid fiber-belt-bars is 25 mm^2^. Table 1 gives the detailed parameters of these fiber-belt-bars, and Figure 1 presents the photos of them.

The mechanical properties of fiber-belt-bars are affected by different braiding techniques. In our work, the fiber-belt-bar was braided with a plain weave. According to the tensile characteristics of the fiber-belt-bar, the tension was mainly borne by the meridional fibers, while the weft fibers bore little load.

A total of four beams were designed, and their corresponding codes were RAL-1, RAL-2, RAL-3 and RHL-1. The tensile reinforcement was made of fiber-belt-bar and steel bar, and AF-K49 and HAC1 were selected as fiber-belt-bars. HRB400 was used for tension reinforcement, and HPB235 was used for stirrup and reinforcement in compression zone. In Table 2, the types and quantity of fiber-belt-bar and reinforcement ratio and other parameters are listed.

Table 3 lists the properties of concrete and different kinds of fiber-belt-bar in concrete beams. The compressive strength of concrete is obtained by compression of cubic standard specimens. Different types of fiber-belt-bars have different tensile moduli, tensile strengths and elongation by tension.

### 2.2. Experimental Methods

**Figure 2:**
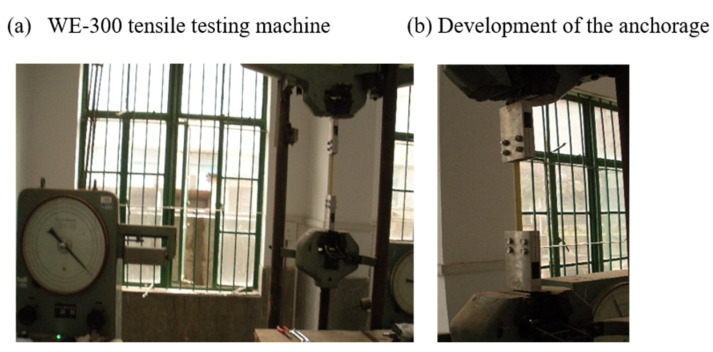
The photos of tensile testing device.

The single fiber-belt-bar in this test are aramid fiber (Kevlar29 and Kevlar49). Hybrid fibers include Kevlar29, Kevlar49, T300 and Kevlar29, T300. Figure 2 shows the tensile test device, mainly using WE300 tensile-testing machine and independently developed anchor. In the process of tensile testing, the deformation of fiber-belt-bars is made by controlling the load. For each time, we increase 10 kN, and the deformation of the fiber-belt-bar was measured after the specimen became stable for 30 s. For each type of fiber-belt-bar, 10 samples with 100 mm in length were used for tensile testing. The specific size of each group is shown in Table 1. 

The main control parameters of the fiber-belt-bar were the type of fiber material and the sectional size of the fiber-belt-bar. The mechanical properties of fiber-belt-bars under different control parameters were studied. This test mainly included the tensile strength, elastic modulus, ultimate elongation and stress–strain curve of the fiber-belt-bar.

**Figure 3:**
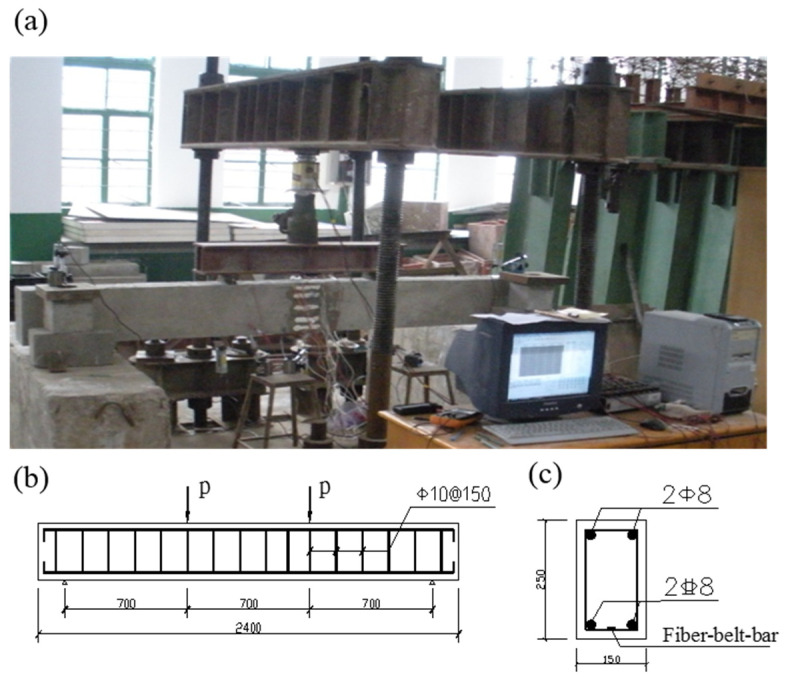
Loading test of the beam. (**a**) Beam loading device diagram, (**b**) Specimen size and reinforcement diagram, (**c**) Reinforcement diagram of cross section of specimen.

The mechanical properties of concrete were tested in accordance with the Chinese standard GBT 50081-2019. The section size of the specimen was B × H = 150 mm × 250 mm, the total length of the specimen was L = 2400 mm, and the span of the beam was 2100 mm. The specific parameters of the specimen are shown in Table 2. Beam loading instruments and equipment mainly include the loading support, distribution beam, jack, sensor, displacement meter, etc. The loading device is shown in Figure 3a. All beams were subjected to two concentrated loads, which were applied by hydraulic jacks. The loading was increased step by step with 5 kN until the cracking appeared. Then, the loading was increasing 10 kN at each step until failure of the specimen.

## 3. Result and Discussion

### 3.1. Experimental Study on the Mechanical Properties

#### 3.1.1. Analysis of the Mechanical Properties of Single Fiber Belt

**Figure 4:**
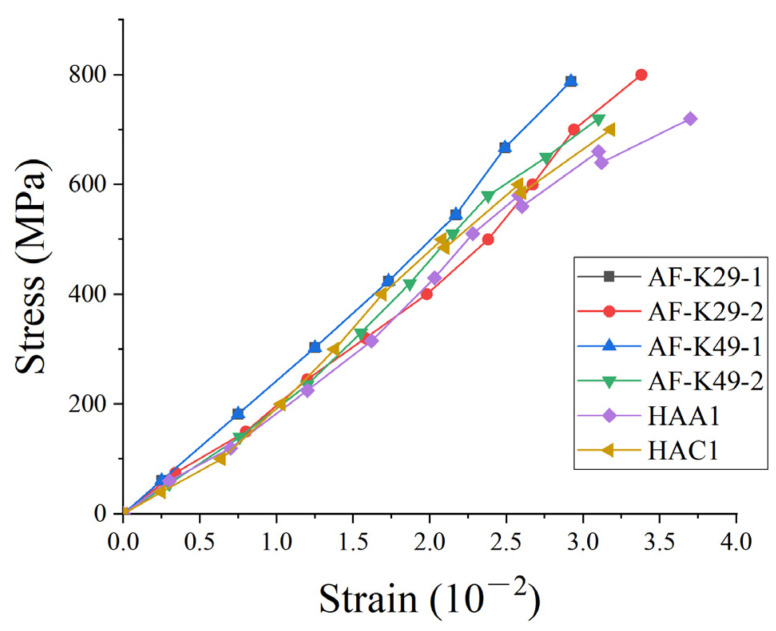
Stress–strain curves of fiber-belt-bars.

According to the test results of tensile failure specimens in the whole process of stress, the stress–strain curve relationship of fiber-reinforced specimens is drawn in Figure 4. The results showed that the stress–strain relationship changed linearly in the whole process from the beginning of loading to the complete failure of the specimen, and the failure of the specimen had no obvious warning. 

The sudden fracture failure of the fiber-belt-bar without an obvious yield stage belonged to brittle failure. The stress–strain relationship curve was “downward convex”, and the strain change rate slowed down with the increase of stress. As the fibrin in the ribbon was bent, the fibrin changed from bent to straightened when the ribbon begins to stretch. The deformation of the filament was mainly its own deformation, and thus the deformation of the fiber-belt-bar became slow.

It can be seen from the figure that the tensile strength of fiber-belt-bars with different materials and sectional sizes was also different. When the sectional size of Kevlar29 was fixed, the tensile strength of Kevlar29 was obviously smaller than that of Kevlar49. Therefore, when different fiber materials were used for braiding, the tensile strength of the fiber-belt-bar was distinctive. The tensile strength of Kevlar29 fiber material was greater than Kevlar49 fiber material; however, the tensile strength of Kevlar29 braided fiber-belt-bar was smaller than Kevlar49 braided fiber-belt-bar, because the loss degree of Kevlar29 fiber was greater than Kevlar49 fiber in the weaving process. 

The tensile strength is also affected by the change of the sectional size of the fiber-belt-bar. In the above tensile test, the sectional size of the fiber-belt-bar was 12 × 3 and 20 × 3, and the tensile strength of the fiber-belt-bar with a section of 20 × 3 was slightly larger than that of the fiber-belt-bar with a section of 12 × 3. When the fiber-belt-bar was loosely braided, the tightness M value was small and the tensile strength of the fiber-belt-bar was large. Otherwise, the tensile strength was low.

As shown in Table 4, the elastic modulus of the fiber-belt-bar was significantly smaller than that of the fiber filament, which was about 20~30% of the elastic modulus of the fiber filament. When the fiber-belt-bar was in the natural state, the meridional filament in the fiber-belt-bar was bent. When the band was subjected to tension, the deformation of the meridional bundle consists of two behaviors, the process of fiber bundle from bending state to straightening state and the stretching deformation process of fiber bundle itself. 

When tensile force was applied to the fiber-belt-bar, two kinds of deformation occurred in the fiber filament of the fiber-belt-bar. However, for the determination of elastic modulus of fiber filament, the deformation of fiber material only included its own deformation. Therefore, when the fiber-belt-bar and the fiber filament were subjected to tension, the fiber-belt-bar produced large deformation under the same load; therefore, the elastic modulus of the fiber-belt-bar was relatively small.

The results indicated that the ultimate elongation of the fiber-belt-bar was larger than that of the fiber filament. When the fiber-belt-bar was braided and the larger the coefficient M was, the closer the fiber was braided. The deformation of the fiber-belt-bar in the stretching process was mainly reflected in the elongation deformation of the fiber bundle itself. When the coefficient M was smaller and the fiber braid was looser, the deformation of the fiber-belt-bar was mainly reflected in the deformation process from bending state to straightening state of the meridional fiber bundle.

#### 3.1.2. Analysis of Mechanical Properties of Hybrid Fiber-Belt-Bars

From the above test analysis, it can be seen that the stress–strain curve of a single fiber strip tensile showed a linear change and there was no obvious yield stage. Therefore, the fiber strip tensile had good ductility unlike the steel bar. According to the theory of fiber composite material, the hybrid braiding of two or more kinds of fiber material had excellent properties that single fiber material did not have, which can improve the stiffness and toughness of fiber material. This test mainly uses aramid fiber and carbon-fiber materials. The common fiber hybrid structures mainly include uniform hybrid, interlayer hybrid, sandwich hybrid and composite hybrid. The fiber-belt-bars in this test mainly adopt uniform hybrid structure to weave fiber materials evenly.

Table 4 lists the average values and standard values of tensile strength of HAA1 and HAC1, respectively. It can be seen from Table 4 that the tensile strength of hybrid fiber-belt-bars was smaller than that of Kevlar49 woven with single fiber material in the above test. In the process of hybrid fiber-belt-bar stretching, the carbon-fiber material first reached the ultimate tensile strain, the carbon-fiber material broken, and then the aramid fiber material broken. Therefore, the tensile strength of the specimen was smaller than that of the fiber-belt-bar sample braided by a single aramid fiber material.

The failure of hybrid fiber-belt-bars in tensile test was obvious, and there was a time sequence of failure between different materials, and thus the stress–strain relationship curve was a folded line. Therefore, the elastic modulus of hybrid fiber-belt-bars is distinctive at different stress stages. Taking Kevlar29 and T300 hybrid braided fiber-belt-bar as an example, when the fiber-belt-bar just began to be affected by tension, its elastic modulus was mainly reflected by Kevlar29 and T300. When the tension reached a certain degree, T300 broken. 

Only Kevlar29 in the fiber-belt-bar was affected by the tension; thus, the elastic modulus at this time was mainly reflected by Kevlar29. For concrete structures with fiber bars, the elastic modulus of origin of fiber bars was mainly considered in the study of the performance of fiber bars, that is, the initial elastic modulus of hybrid braided fiber bars was studied. Based on the above analysis, the hybrid braided fiber-belt-bar had a certain ductility compared with the single fiber-belt-bar; however, its tensile strength decreased. With the increase of carbon fiber content, the decrease degree of tensile strength of hybrid fiber-belt-bar increased.

### 3.2. Flexural Test on Normal Section of Fiber Reinforced Concrete Beam

#### 3.2.1. Characteristics of Cracking Load and Crack Distribution

**Figure 5:**
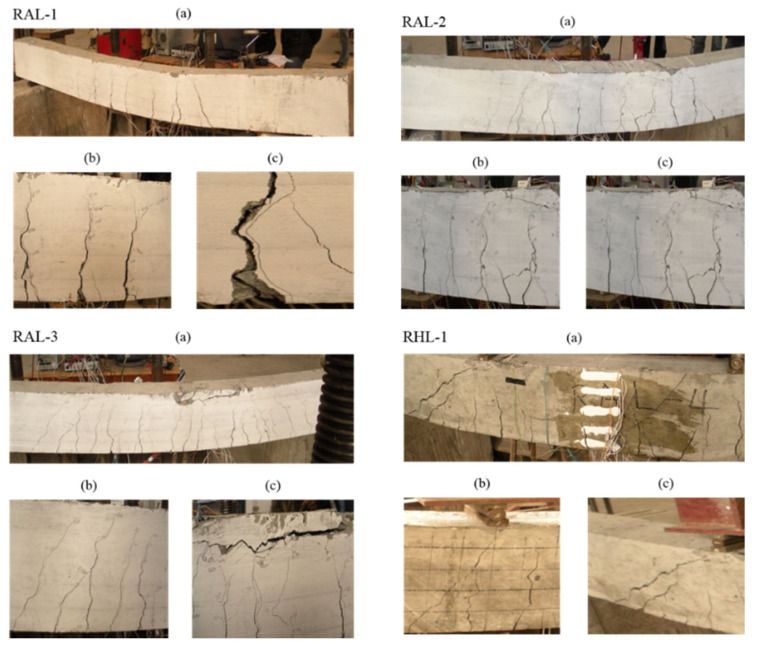
Test photos of RAL-1, RAL-2, RAL-3 and RHL-1. (**a**) Beam bending deformation, (**b**) Distribution of major fractures, (**c**) Crushing failure of concrete.

**Figure 6:**
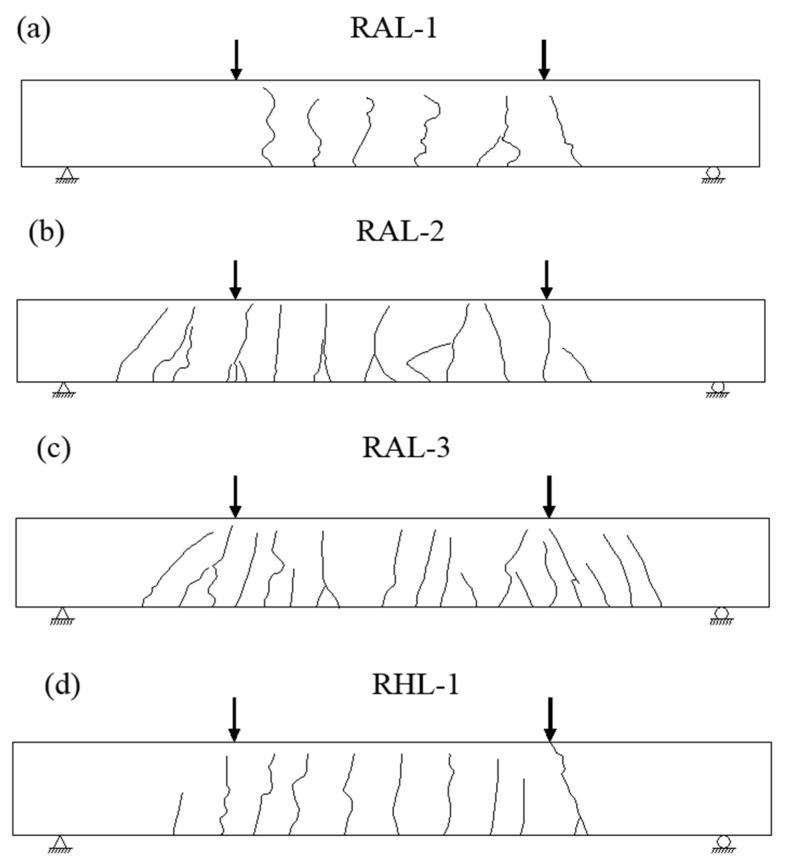
Crack shape and distribution of the test beam. (**a**) RAL-1, (**b**) RAL-2, (**c**) RAL-1, (**d**) RHL-1.

Figure 5 shows the crack distribution and characteristics of RAL-1, RAL-2, RAL-3 and RHL-1, respectively. The sketch of crack distribution and characteristics of each test beam under load is redrawn in Figure 6. The results showed that the development of each fracture was not synchronous. At the beginning of loading, the crack on the side of concrete extends up faster; however, when it reached a certain height, the crack extension height changed slowly. 

At the same time, the crack spacing of the component with different reinforcement ratio was different. The amount of reinforcement in the tensile zone of the four test beams was the same; however, the number of fiber-belt-bars was distinctive. When there were more fiber-belt-bars in the tension zone, the width of cracks was smaller and the number of cracks was larger. The crack width and the average crack spacing were larger when the fiber-belt-bars were less in the tension zone.

**Figure 7:**
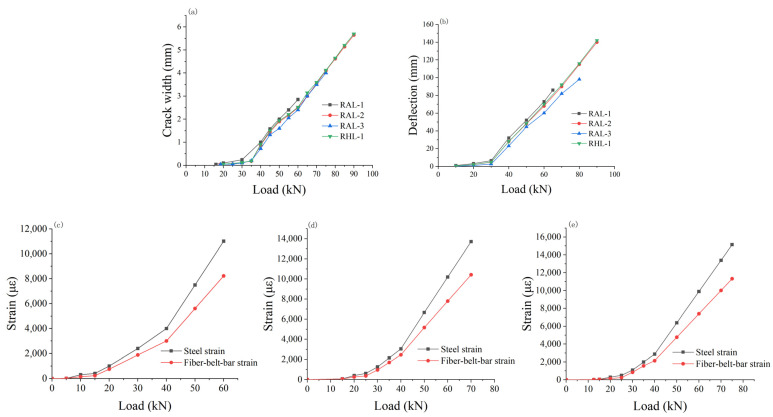
The test related data results. (**a**) Maximum crack width curves of specimens under different loads, (**b**) Deflection curves of specimens under different load, (**c**) Stress-strain curves of specimen RAL-1 under different loads, (**d**) Stress-strain curves of specimen RAL-2 under different loads, (**e**) Stress-strain curves of specimen RAL-3 under different loads.

Figure 7a shows the development of the maximum crack width of each test beam with the change of load. When the load was applied to about 30 kN, the crack-width–load relationship curve had an obvious “inflection point”. According to the above strain analysis of the fiber belt and steel bar in the tensile zone under different loads, when the load reached about 30 kN, the steel bar entered the yield stage, because the crack width in the tensile zone was mainly related to the applied load and the bond between concrete, fiber belt and steel bar. 

When the reinforcement reached the yield, the tensile force of the reinforcement remains unchanged, and the increase of the bending moment of the beam section was mainly realized by the increase of the tensile force of the fiber-belt-bar. With the increase of external load, a little slippage occurred in the fiber-belt-bar reinforcement, and thus the bond between the fiber-belt-bar reinforcement and concrete decreased. According to the stress mechanism of cracks, it can be considered that the cracks in the tensile zone expanded faster.

When the ratio of reinforcement used in the beam was different, the width of crack generated was distinctive. The maximum crack width increased with the increase of reinforcement ratio. As can be seen from Figure 7a, the maximum crack width of RAL-1 was larger than that of RAL-3 under the same load. When the applied load was small, the fiber-belt-bar reinforcement, steel bar and concrete kept close bond. When the reinforcement ratio was large, the crack width of the tensile zone was small. 

With the increase of the load, the bond property between the tensile bar and concrete decreased, and the crack width in the tensile zone was less affected by the bond property of steel bar, fiber-belt-bar bar and concrete. Therefore, the difference of crack width in the tensile zone of the beam was not obvious even when the ratio of fiber-belt-bar reinforcement was increased, indicating the influence of fiber-belt-bar reinforcement on the crack of the beam was limited. In the design of the beam, the crack width can control the design bearing capacity of members, which can make the structure had a greater degree of safety.

Table 5 lists the cracking load, ultimate load and failure of components. When the beam adopted different reinforcement ratio, its cracking load changed. With the increase of the section reinforcement ratio, its cracking load also increased. It can be found from the cracking load difference of the four test beams, the maximum difference was 16%. The reinforcement ratio of the section had an effect on the cracking load of beams; however, the effect was limited. When the reinforcement ratio of fiber-reinforced concrete beam was different, its ultimate load was also distinctive. With the increase of reinforcement ratio, the ultimate load increased, which was similar to the reinforced concrete beam. 

The failure of concrete flexural specimens with fiber reinforcement can be divided into tensile failure of fiber reinforcement and crushing failure of concrete in compression zone. However, the shear failure of fiber-belt-bar reinforcement was not the ideal failure mode of beam normal section bending, because the shear force of beam was large, which lead to the shear failure of fiber-belt-bar reinforcement. This kind of phenomenon should be avoided in the future design of beams.

#### 3.2.2. Strain Changes of Tensile Bars

Figure 7c–e depicts the changes of steel bars and fiber-belt-bars in the tensile zone of RAL-1, RAL-2 and RAL-3 as a result of loading. When the initial load was applied, the strain difference between the steel bar and the fiber-belt-bar was not obvious; however, with the increase of the load, the strain difference between the steel bar and the fiber-belt-bar increased. Under the same load, the strain of reinforcement was greater than that of fiber-belt-bar reinforcement. 

At the initial stage of loading, the bond between fiber-belt-bar reinforcement, steel bar and concrete were kept close, and the deformation between fiber-belt-bar reinforcement and steel bar was basically the same, and the deformation difference between them was relatively small. With the increasing of the load, the bond between the tensile bar and concrete changes, and the strain of the fiber-belt-bar bar and steel bar increased rapidly. When the steel bar in tension zone changed from the elastic stage to plastic stage, it can produce large strain even if a small load is applied. In this stage, the strain of reinforcement was greater than that of fiber-belt-bar reinforcement under the same load.

#### 3.2.3. Load-Deflection Curve

Using three-point loading, the mid-span deflection changes of the beam with pure bending section and shear force and bending moment coexist were obtained, as shown in Figure 7b. The results showed that the load deflection curve of fiber-reinforced concrete beams can be divided into three stages: from the beginning of loading to the cracking of concrete in the tension zone of the beam, from the cracking of concrete to the yielding of reinforcement, and from the yielding of reinforcement to the failure of the specimen. The turning point corresponds to the cracking load of the concrete beam and the corresponding load when the reinforcement in the tensile zone of the beam reached the yield.

There was a linear relationship between the deflection and load in each stage. The analysis of the stress of the steel bar and the fiber-belt-bar bar in the beam section showed that, with the increase of the load, the tensile steel bar entered the yield stage first, while the fiber-belt-bar bar was still in the linear elastic stage. With the further increase of the load, the tensile force of the reinforcement basically remains unchanged, and the increase of the bending moment of the section was mainly realized by the tensile fiber-belt-bars.

The relationship between deflection and load of reinforced concrete beams was related to the proportion of reinforcement and fiber reinforcement in the tensile zone. Before the cracking of the beam specimen, the main factors affecting the deflection of the beam were the size of the beam section, the span of the beam, the strength grade of the concrete and the way of loading, etc., while the influence of the ratio of reinforcement was relatively small, showing the characteristics of a plain concrete beam. 

The deflection of beam specimens from concrete cracking to the steel yielding stage is mainly related to the ratio of tensile reinforcement in the tensile zone. The larger the ratio of reinforcement was, the smaller the deflection was. With the further increase of the load, the reinforcement yielded and the subsequent increase of the load was mainly borne by the fiber-belt-bar reinforcement in the tension zone. 

Therefore, the deflection deformation of the beam was mainly affected by the ratio of fiber-belt-bar reinforcement in the tensile zone. The higher the ratio of fiber-belt-bar reinforcement in the tensile zone, the smaller the deflection deformation of the beam. According to the reinforcement of the four beams, the same reinforcement was used in the tensile zone of the beams. The *As* was the same; however, the cross-section area of fiber-belt-bar reinforcement *A_f_* was different. Under the same load, the deflection change was not very obvious. From the overall consideration of the fiber-reinforced concrete beam, it can be considered that the fiber-reinforced concrete beam contributed little to the deflection of the beam specimen. 

When the coefficient *K_f_* was large, the beam was mainly the fiber-reinforced concrete beam, and its deflection was obviously larger than that of the reinforced concrete beam. However, when *K_f_* was small, the beam mainly showed the characteristics of reinforced concrete beam and the deflection was reduced. Based on the above analysis, it can be concluded that for different values of *K_f_*, the deflection size was also different. When the deflection size of the beam needed to be changed, the value of *K_f_* can be changed, that is, it was also an effective method to consider changing the proportion relationship between the fiber-belt-bar and steel bar.

### 3.3. Improved Calculation of Crack Width and Deflection of Fiber Reinforced Concrete Beams

#### 3.3.1. Derivation of Calculation Formula for Crack Width

It was known from the above experimental analysis that the crack mechanism of fiber-reinforced concrete beams was similar to that of reinforced concrete members subjected to bending. Combined with the characteristics of fiber-reinforced concrete beams: (i) Fiber-reinforced concrete beams and steel bars borne tensile force together. (ii) The elastic modulus and bond performance of two kinds of tensile bars were different; thereby, the calculation formula of crack width of fiber-reinforced concrete beams was derived [23].

According to the above analysis, the main factors affecting the crack width of fiber-reinforced concrete beams were: the stress or strain of tensile bars, the thickness of concrete protective layer, the bond between tensile bars and concrete, the binding effect of tensile bars on local concrete, the area and number of tensile bars. According to the Gergely–Lutz formula for crack width of reinforced concrete beams adopted in the Canadian code and ACI code [21], the calculation formula for maximum crack width of reinforced concrete beams with fiber bars under short-term load is given as follows:

(1) $$\omega_{\max}=k_{g}\cdot \varepsilon_{}\cdot \beta^{\prime }\cdot \sqrt[{3}]{d_{c}A}\times 10^{-6}$$

where $d_{c}$ is the distance from the center of the bottom row of the beam to the edge of the concrete tensile zone. A is the average effective area of the tensile concrete around each tensile bar. ε is the strain of the tension bar under the action of bending moment. $\beta^{\prime }$ is the strain gradient. $k_{g}=f(\rho_{s},\rho_{f},\upsilon_{s},\upsilon_{f})$ is a function that reflects the influence of bond property of tensile bars on crack width in fiber-reinforced concrete beams. $\nu_{s}$ is the relative bond coefficient of steel bars, which is 1 for deformed steel bars and 0.7 for smooth round steel bars. $\nu_{f}$ is the relative bond coefficient of fiber-belt-bar, which is =0.52. $\rho_{s}$ and $\rho_{f}$ are the reinforcement ratio of steel bar and fiber-belt-bar bar, respectively.

As the bond force of the tensile bar is related to the contact area of the fiber-belt-bar bar. For the convenience of calculation, the section shape of the fiber-belt-bar can be equivalent to a circle. According to the relation between area and diameter, the cross section area of the tension bar has a square relation with its diameter. The crack width was a function of the reinforcement ratio. With the increase of the reinforcement ratio, the crack width decreased, and there was a certain relationship between the reinforcement ratio and the section area of the tensile reinforcement. Therefore, the variables in the *k_g_* expression can be regarded as only two, $\nu_{s}\sqrt{\rho_{s}}$ and $\nu_{f}\sqrt{\rho_{f}}$. The *k_g_* can be computed by introducing the variability:

(2) $$y=\frac{1}{\nu_{s}\sqrt{\rho_{s}}+\nu_{f}\sqrt{\rho_{f}}}$$

(3) $$k_{g}=k_{1}y$$

where *k*_1_ is an undetermined constant, which needs to be determined by the test.

According to the maximum crack width $\omega_{\max}$ measured by each specimen in the test and the strain value ε of the tension bar, the corresponding value of $k_{g}$ can be obtained from Equation (1). The specific value can be seen in Table 6. The y can be calculated according to the ratio of reinforcement of the specimen and the relative bond coefficient of steel bar and fiber-belt-bar reinforcement. Under short-term load, the maximum crack width of the beam can be expressed as:

(4) $$\omega_{\max}=\frac{0.43}{\nu_{s}\sqrt{\rho_{s}}+\nu_{f}\sqrt{\rho_{f}}}\varepsilon \beta^{\prime }\sqrt[{3}]{d_{c}A}\times 10^{-6}$$

In order to verify the rationality of the proposed formula, the experimental and theoretical values of the beam were compared and analyzed. Table 6 lists the comparison between the measured and theoretical crack width values of the specimens under the loading of 20, 40 and 60 kN, respectively.

By analyzing the comparison between the calculated value and the test value of the crack width in the tensile zone of the test beam under the load of 20, 40 and 60 kN, Equation (4) can better predict the maximum crack width in the tensile zone of the fiber-reinforced concrete beam under the load.

**Figure 8:**
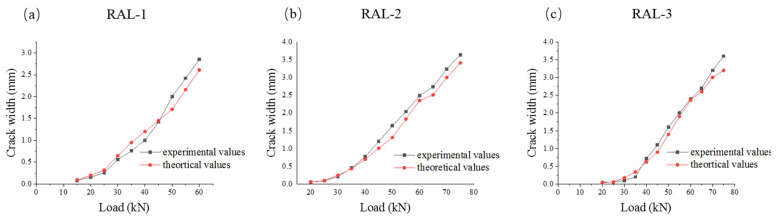
Comparison between experimental and theoretical values of crack width of test beam. (**a**) RAL-1, (**b**) RAL-2, (**c**) RAL-3.

In order to describe the stress process and crack development of beams more clearly, Figure 8 describes the relationship between measured and theoretical values of maximum crack width of specimens RAL-1, RAL-2 and RAL-3 during the whole process of loading. Figure 8 can reflect the deviation degree between the measured value and the theoretical value of the crack width of the test beam in the whole process of stress. The research on the maximum crack width of ordinary specimens mainly considered the calculation of the maximum crack width under normal service load. The results showed that the theoretical value was in good agreement with the measured value.

#### 3.3.2. Calculation of Deflection of Flexural Component under Short-Term Load

For uniform elastic beam, the bending stiffness remains unchanged at each section, and the deflection can be calculated by structural mechanics or finite element method. But for reinforced concrete beams and fiber-reinforced concrete beams, cracks leaded to the reduction of the bending stiffness of the cross section, and there was a certain distance between cracks. The concrete also bears part of the tension between the cracks, which increased the stiffness of the components. 

Therefore, even in the pure bending section with the same bending moment, the stiffness of each section was not equal, and the stiffness at the crack section was the smallest. These factors bring great difficulties to deflection calculation, and thus the key to deflection calculation was to determine the bending stiffness EI. In consideration of the differences in bond properties between fiber-belt-bar bars, steel bars and concrete in the tensile zone of beams, the coefficients *α* and *β* were introduced to modify *I_e_* formula in the code. The expression of effective moment of inertia of fiber-reinforced concrete beam can be further simplified [22].

When $M_{k}<M_{cr}$:

(5) $$I_{e}=I_{g}$$

When $M_{k}\geq M_{cr}$:

(6) $$I_{e}=\beta I_{g}{\left(\frac{M_{cr}}{M_{k}}\right)}^{3}+\alpha I_{cr}\left[1-{\left(\frac{M_{cr}}{M_{k}}\right)}^{3}\right]\leq I_{g}$$

where *M_k_* is the standard bending moment value calculated according to the standard combination of load effects. *M_cr_* is the cracking moment of fiber-reinforced concrete beam member.

The *I_g_* is the moment of inertia of section of concrete beam member with fiber reinforcement before cracking.

(7) $$I_{g}=bh^{3}/12\left[1+1.7(\alpha_{s}-1)\rho_{s}+1.7(\alpha_{f}-1)\rho_{f}\right]$$

where $\alpha_{s}=E_{s}/E_{c}$ and $\alpha_{f}=E_{f}/E_{c}$ are the ratio of the elastic modulus between steel bar and fiber reinforcement and concrete, respectively.

**Figure 9:**
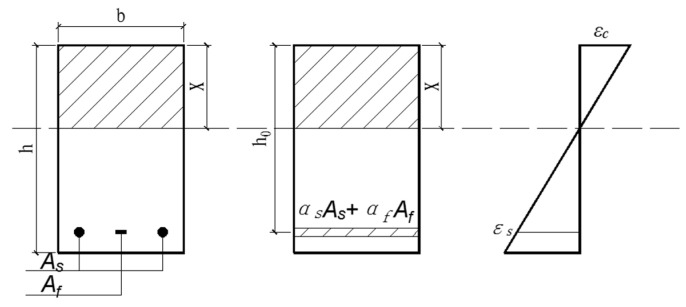
Schematic diagram of conversion section.

After cracks appeared in the member, the effect of concrete in the tensile zone was ignored, and only the fiber-belt-bar and steel bar borne the tensile effect in the tensile zone. The area of fiber-belt-bar and steel bar can be converted into the concrete area of the same height, and the moment of inertia *I_cr_* of the cracked section can be calculated. The converted cross section is shown in Figure 9.

For the single-bar rectangular section shown in Figure 9, the converted section area A_0_ and the converted section moment of inertia *I_cr_* are:

(8) $$A_{0}=bx+\alpha_{s}A_{s}+\alpha_{f}A_{f}$$

(9) $$I_{cr}=\frac{1}{3}bx_{}^{3}+(\alpha_{s}A_{s}+\alpha_{f}A_{f}){\left(h_{0}-x_{}\right)}^{2}$$

where *h*_0_ is the effective stiffness of the tension bar. *x* is the height of the compression zone of cracked section concrete.

According to the neutral axis of the cracking section of the bending specimen, through the centroid axis of the converted section, namely, the static moment of the compression zone to the neutral axis is equal to the static moment of the tension zone to the neutral axis. The formula for *x* is as follows:

(10) $$\frac{1}{2}bx_{}^{2}=(\alpha_{s}A_{s}+\alpha_{f}A_{f})(h_{0}-x_{})$$

(11) $$x=\frac{\sqrt{{\left(\alpha_{s}A_{s}+\alpha_{f}A_{f}\right)}^{2}+2bh_{0}(\alpha_{s}A_{s}+\alpha_{f}A_{f})}-(\alpha_{s}A_{s}+\alpha_{f}A_{f})}{b}$$

*β* is the reduction coefficient, which is related to the elastic modulus of steel bar, fiber-belt-bar bar and bond property. For reinforced concrete beam, desirable *β* = 1. When *K_f_* = 1, *β* = *β_f_* is assumed for pure fiber-reinforced concrete beams. For fiber-reinforced concrete beams, *β* can be obtained by interpolation in the following formula:

(12) $$\beta =\beta_{f}+(1-\beta_{f})\sqrt{A_{s}/(A_{s}+A_{f})}$$

(13) $$\beta_{f}=\alpha_{b}(1+E_{f}/E_{s})$$

where α_b_ is the coefficient related to the bond performance between fiber-belt-bar reinforcement and concrete, *α_b_* = 0.5, and substituted into Equation (13), *β_f_* = 0.56. *α* in Equation (6) is another reduction coefficient, and the value of α can be determined according to the test of FRC beams. Through data fitting, *α* = 0.26.

**Figure 10:**
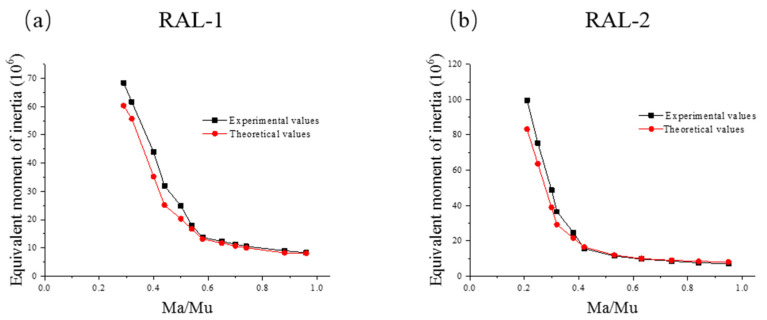
Comparison between experimental and theoretical values of equivalent moment of inertia of beam. (**a**) RAL-1, (**b**) RAL-2.

In order to verify the rationality of the effective moment of inertia mentioned above, the section stiffness of RAL-1 and RAL-2 were calculated and compared with the measured values. Figure 10 compares the experimental and theoretical equivalent moment of inertia of experimental beams RAL-1 and RAL-2. The results showed that when the applied load was small, there was a certain difference between the measured value and the theoretical value of the beam section moment of inertia, and the two curves were in good agreement with the increase of the applied load. Figure 10 also showed that the effective moment of inertia of beams can be well predicted by using Equation (6).

Finally, the short-term stiffness B_s_ of the beam can be expressed by the product of the elastic modulus *E_c_* of concrete and the effective moment of inertia *I_e_* of the section. Using structural mechanics or finite element method, the deflection of the specimens under the short-term load can be calculated.

In order to verify the rationality of the formula, it was also necessary to compare and analyze the measured values of the test components with those of the above theoretical equation. In addition, the deflection calculation of the component was mainly concerned with its deflection in the stage of normal service load; therefore, the deflection calculation of the component was mainly concerned with its calculation value under normal service load.

When the deflection calculation formula proposed above was applied, the ratio between the test value and the theoretical value ranged from 0.77 to 1.06, thus, indicating that the deflection test value of the test beam was close to the theoretical value under the loading of 20, 40 and 60 kN. In Figure 10, the relationship between experimental and theoretical values of effective moment of inertia of concrete beams with fiber bars can reflect the relationship of deflection of beams under short-term load to a certain extent. When the relationship between the two curves was close, it means that the deflection test value of the beam was in good agreement with the theoretical value. Based on the above two aspects, the deflection calculation formula can be used to predict the deflection of fiber-reinforced concrete beams.

## 4. Conclusions

In this paper, we mainly made an experimental study and theoretical analysis on the mechanical properties and the mechanical properties of fiber-reinforced concrete beams. By replacing part of tensile steel bar with fiber-belt-bar, the advantages of fiber-belt-bar and steel bar can be brought into play simultaneously, and the durability of concrete structure can be improved. Experimental studies on six groups of tensile specimens and four fiber-reinforced concrete beams were performed. The main conclusions are as follows:

1. The tensile strength of the fiber-belt-bars was lower than that of the fiber filament. The tensile strength of the fiber was generally about 30% of the tensile strength of the fiber filament. The tensile failure of a single fiber-belt-bar was a fracture failure, and the stress–strain curve changed linearly before failure. However, the stress–strain relationship of hybrid fiber-belt-bars had strain mutation, and the stress–strain relationship showed a broken line change.
2. The coefficient in the formula for calculating the maximum crack width of reinforced concrete beams proposed in ACI standard was adjusted appropriately so that the formula can meet the requirements of the maximum crack width of concrete beams with fiber bars. By comparing the theoretical calculation value of the formula with the measured value of the test, the crack calculation formula proposed in this paper can predict the maximum crack width of the beam under various loads.
3. The effective moment of inertia *I_e_* in the ACI standard was modified by introducing the coefficients *α* and *β*. The coefficient α was derived from the test beam, and the coefficient *β* was derived from the elastic modulus and relative bond coefficient of the fiber-belt-bar. Through data comparison, we concluded that the deflection calculation formula proposed in this paper can better predict the deflection size of the beam. According to the bending characteristics of fiber-reinforced concrete beams under a normal service load, the maximum crack width or deflection limit may be reached before the test beams reached the ultimate state of bearing capacity. Therefore, the beam may be controlled by the limit state of normal service or the limit state of the bearing capacity with a different reinforcement ratio, section size and coefficient *K_f_.*

## Figures and Tables

**Table 1:** Details of the fiber-belt-bar parameters.

Sample Code	Materials	Representation Area (mm^2^)	Actual Cross-Sectional Area (mm^2^)
AF-K29-1	AFRP (Kevlar29)	12 × 3	16.5
AF-K29-2	AFRP (Kevlar29)	20 × 3	25
AF-K49-1	AFRP (Kevlar49)	12 × 3	16.5
AF-K49-2	AFRP (Kevlar49)	20 × 3	25
HAA1	80% K29 + 10% K49 + 10% T300	20 × 3	25
HAC1	80% K29 + 20% T300	20 × 3	25

**Table 2:** Details of the beam specimen parameters.

Sample Code	Fiber Types	Number of Fiber-Belt-Bar (Root)	*ρ_f_* (%)	*A_s_* (mm^2^)	*ρ_s_* (%)	*K_f_*
RAL-1	AF-K49-2	1	0.18	100.48	0.3	0.37
RAL-2	AF-K49-2	3	0.54	100.48	0.3	0.64
RAL-3	AF-K49-2	4	0.73	100.48	0.3	0.72
RHL-1	HAC1	3	0.54	100.48	0.3	0.64

Note: *A_f_*: Cross section area of fiber with reinforcement; *ρ_f_*: fiber-belt-bar reinforcement ratio; *A_s_*: Reinforcement section area; *ρ_s_*: Ratio of steel reinforcement; *K_f_*: *A_f_*/(*A_f_* + *A_s_*).

**Table 3:** The material properties of beam specimens.

Materials	Concrete	AF-K49-2			HAC1		
Performance Indictors	The Compressive Strength *f_cu_* (MPa)	Tensile Modulus *E_af_* (GPa)	Tensile Strength *f_fu_* (MPa)	Elongation *ε_afu_*	Tensile Modulus *E_af_* (GPa)	Tensile Strength *f_fu_* (MPa)	Elongation *ε_afu_*
Average values	48	24	946	0.039	21	783	0.037
Standard values	43	—	922	—	—	760	—

**Table 4:** Elastic modulus and elongation of fiber-belt-bars.

Sample Code	AF-K29-1	AF-K29-2	AF-K49-1	AF-K49-2	HAA1	HAC1
Effective cross section (mm^2^)	16.5	25	16.5	25	25	25
Average values (MPa)	873	874	921	946	845	783
Standard values (MPa)	829	828	871	922	824	760
standard deviation σ	27	28	31	24	13	14
Modulus of elasticity (GPa)	26	22	26	24	20	21
Elongation (%)	3.36	3.97	3.54	3.94	5.35	5.50

**Table 5:** The cracking, ultimate load and failure of test beams.

Sample Code	Cracking Load (kN)	Ultimate Load (kN)	Damage
RAL-1	13.5	68	Tensile failure of fiber-belt-bar
RAL-2	15.5	95	Concrete crushing
RAL-3	16	80	Concrete crushing
RHL-1	15	95	Shear failure of fiber-belt-bar

**Table 6:** The measured values of crack width of test beam.

Sample Code	$\rho_{\mathrm{fs}}\;(\%)$	$\rho_{0}\;(\%)$	$K_{f}$	Mean Crack Width		
				20 kN	40 kN	60 kN
				Width (mm)	Width (mm)	Width (mm)
RAL-1	0.49	0.18	0.37	0.16	1.00	2.85
RAL-2	0.85	0.54	0.64	0.06	0.77	2.5
RAL-3	1.03	0.72	0.72	0.05	0.72	2.4
RHL-1	0.85	0.54	0.64	0.06	0.79	2.52

## Data Availability

Data sharing is not applicable.
